# Natural‐Light‐Initiated 3D Macro Zigzag Architecture of Graphene‐Reinforced Polystyrene for Gravity‐Driven Oil and Water Separation

**DOI:** 10.1002/gch2.201800040

**Published:** 2018-09-02

**Authors:** Nadeem Baig, Tawfik A. Saleh

**Affiliations:** ^1^ Chemistry Department King Fahd University of Petroleum & Minerals Dhahran 31261 Saudi Arabia

**Keywords:** 3D porous network, energy, graphene, natural light harvesting, polymers

## Abstract

Superhydrophobic 3D robust materials are introduced for the separation of hexane and water. For the first time, novel 3D zigzag polystyrene on graphene‐incorporated polyurethane (3D zz‐PS/GR/PU) is prepared using exclusively natural sunlight without any chemical initiator. The zigzag polystyrene growth is accomplished by polymerizing the styrene vapors. The natural sunlight provides a compact 3D zz‐PS/GR/PU material with superoleophilic and hydrophobic channels that allow for the rapid passage of oil, whereas water is entirely prevented from passing. The 3D zz‐PS/GR/PU compact channels are transformed into the compressible material by treating them with toluene without affecting the hydrophobicity of the material. The 3D zz‐PS/GR/PU displays a high‐water contact angle of approximately 150°. The developed materials are characterized by FTIR, SEM, and BET. The graphene incorporation makes surface area of the 3D zz‐PS/GR/PU substantially large compared with PU. It is improved from 15 to 67 m^2^ g^−1^. The pore size of the adsorption and desorption in the 3D zz‐PS/GR/PU is also reduced from 354 and 352 Å to 34 and 33 Å. The 3D zz‐PS/GR/PU satisfies the requirement of high‐demanding superhydrophobic materials, like a low‐cost fabrication process, reusability, and tunability. This strategy can trigger large‐scale production with a controlled morphology.

## 1 Introduction

Rapid industrialization and the fast‐growing world population have resulted in high demand for energy. Oil spillage incidents have become common due to the frequent oil movements across the world. In history, the Deep‐water Horizon oil spill (2010) in Mexico was considered to be a major oil spillage accident in marine water. Five million barrels of oil were released, and the deaths of 11 people were reported due to this accident. Another incident in North Dakota, the USA in 2016 caused the release of 4200 barrels of oil.^1^ The waste from industry also contains a huge quantity of oil which causes severe water pollution. Oil pollution is a critical threat to living organisms and their healthy sustainability in the ecosystem.^2^, ^3^, ^4^ However, oil removal from water is a major challenge to maintain a clean aquatic system due to the continuing increase in oil pollution.^5^, ^6^

Oil–water separation is of great interest to the researchers due to its economic, social, and environmental significance.^7^, ^8^ One of the crucial factors in the performance of the materials used for oil and water separation is their wettability.^9^ Superhydrophobic surfaces can be prepared through layer by layer assembly,^10^ coating,^10^ the dip coating method,^11^ the drop coating method,^12^ chemical vapor deposition,^13^ and the sol–gel method.^1^, ^14^ Various supports were also used to develop superhydrophobic surfaces including mesh,^15^ sponges, foams,^16^, ^17^ and fabrics.^18^ The hydrophobic surfaces are generated using a range of materials such as metal, metal oxide nanoparticles,^18^ polymers^19^ and carbon nanomaterial.^20^, ^21^

Recently, 2D graphene and its derivatives have received extraordinary attention due to their unique physicochemical properties. Graphene and its derivatives are extensively used to improve the chemical, electrical, mechanical, and thermal behavior of materials.^22^, ^23^, ^24^, ^25^, ^26^, ^27^ Graphene, apart from its excellent electrochemical properties, also possesses amazing hydrophobic properties.^11^, ^28^ The hydrophobic behavior of graphene was exploited to fabricate the hydrophobic materials for the separation of oil from water.^11^, ^29^ Graphene‐based hydrophobic foam can be synthesized by a simple dip coating method using a 3D polymer skeleton. Graphene oxide (GO) was used as a precursor to obtain graphene. The graphene‐coated foam displayed good hydrophobicity.^30^ Spongy graphene was obtained without any support through the hydrothermal method by enclosing the GO into a sealed reactor of the desired shape and subjected to heat treatment for 24 h at 180 °C. The obtained graphene gel was freeze‐dried for 48 h to obtain spongy graphene. It has a high tendency for petrol absorption, as well as fat.^31^ The magnetic foam was obtained by the combination of magnetic nanoparticles (Fe_3_O_4_) and the graphene/reduced graphene oxide (rGO). The introduction of magnetic properties into foam facilitates the facile movement and removal of foam after oil absorption.^20^ Similarly, a magnetic nanoparticle functionalized free standing rGO foam was synthesized by the hydrothermal method.^32^

In most of the cases, rGO is obtained from the GO. The synthesis and the handling of the GO are easy, cost effective and it can be produced at large scale.^33^ GO has a strong hydrophilic character and its hydrophobicity is increased by reducing it.^34^ However, the GO cannot be fully reduced and some oxygen functionalities still remain on the surface of the rGO^28^ which reduces the hydrophobicity of the rGO.

To overcome this drawback, the rGO and polystyrene 3D network synthesized on polyurethane using natural sunlight initiated polymerization in a confined glass reactor for oil and water separation. The rGO in the material is not enough to impart superhydrophobic behavior due to the preservation of the oxygen functionalities in the reduction process. Herein, efforts are made to construct a 3D superhydrophobic porous network with the help of polyurethane and polystyrene. Polystyrene is a good hydrophobic material but the introduction of the porous network into the polystyrene is a challenging task. In this work, the porous 3D network of polystyrene and graphene was synthesized through a green methodology that displayed excellent mechanical stability with a superhydrophobic behavior. The polymerization process was carried out under natural sunlight which provided a green, nonhazardous and cost‐effective route for the bulk production of the superhydrophobic material with a better uniform controlled morphology. A zigzag‐shaped growth of polystyrene on the surface of the GR/PU was observed which might be helpful to provide a better surface area for the efficient separation of oil from water. The synthesized porous superhydrophobic material can be shown to be a better choice for the removal of the bulk contamination of nonpolar organic solvents and oils from water.

## 2 Results and Discussion

### 2.1 Mechanism and Process of Growing 3D zz‐Polystyrene into GR/PU

The synthesized hydrophobic material 3D zz‐PS/GR/PU is a combination of graphene, polystyrene, and polyurethane. The incorporation of graphene into the polyurethane network provided a large surface area and also contributed to improving the mechanical properties of the material. The midway hanging of the GR/PU into the glass reactor assisted with achieving uniform growth of PS in 3D zz‐PS/GR/PU. The glass reactor played a crucial role in the formation of the 3D zz‐PS/GR/PU. The glass reactor walls were transparent and permitted the sunlight radiation to enter the reactor through it. The synthesis process of 3D zz‐PS/GR/PU was completed in two steps. The styrene liquid cannot approach directly to the GR/PU because it is hanging midway in the middle of the reactor. The sunlight radiation entered into the transparent glass reactor that provided the heat to vaporize the volatile styrene and might also have established equilibrium between the styrene vapors and the liquid styrene in the reactor. Vapors can move freely in the free space of the reactor and some of them stayed and passed through the porous network of the GR/PU. Simultaneously, the second step of polymerization was started. During this step, the polymerization process of styrene was initiated by the natural light. In this polymerization step, the styrene vapors started to convert into polystyrene and further stimulated the styrene liquid to vaporize. This process was continued until the polymerization process was completed.

Step 1: Vaporization


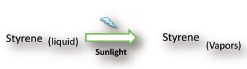


Step 2: Polymerization


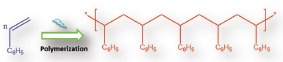


**Figure 1:**
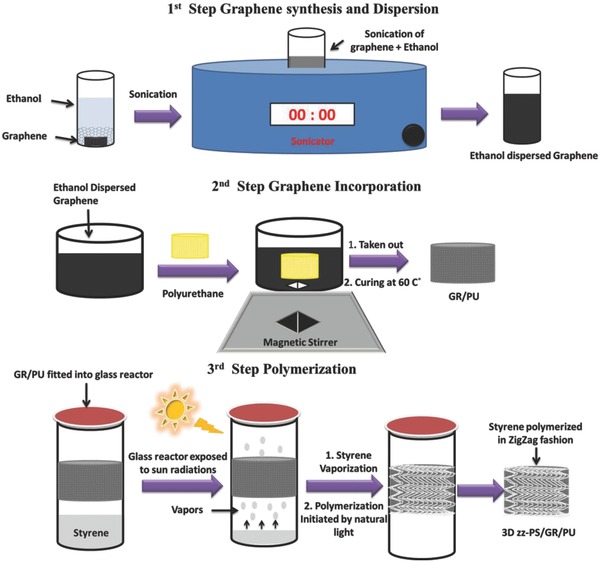
The schematic representation of the fabrication of 3D zz‐PS/GR/PU for oil and water separation.

The critical observation was made, after the polymerization of polystyrene on the pure polyurethane, that the interior of the PS/PU became fragile. It was shattered into small pieces by touching its surface. However, the incorporation of graphene into PU enhanced the growth of the polystyrene and the surface was more mechanically stable. The growth of polystyrene from the vapors of styrene is more interesting and provided a particular zigzag pattern of polystyrene that was porous in nature. This methodology might provide a better opportunity to combine the intrinsic characteristics of the polyurethane, graphene and the polystyrene for efficient utilization in various targeted fields. The fabrication process for the 3D macro zigzag architecture of graphene‐reinforced polystyrene is illustrated in **Figure** **1**.

### 2.2 Surface Morphology and FTIR Study of the Materials

**Figure 2:**
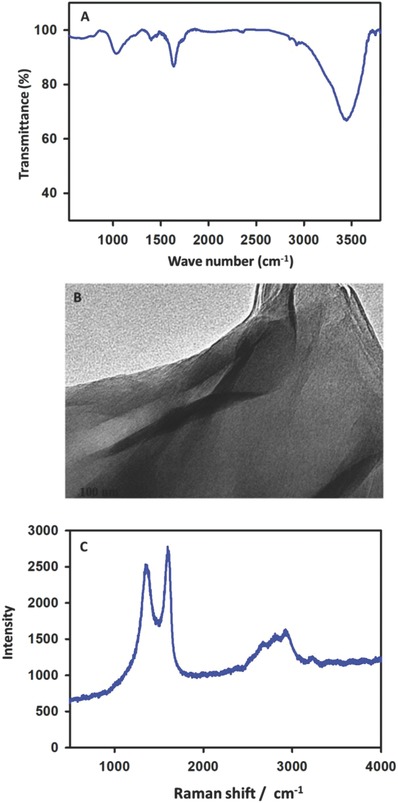
A) FTIR spectra of graphene, B) TEM image of graphene, and C) Raman spectra of graphene.

**Figure 3:**
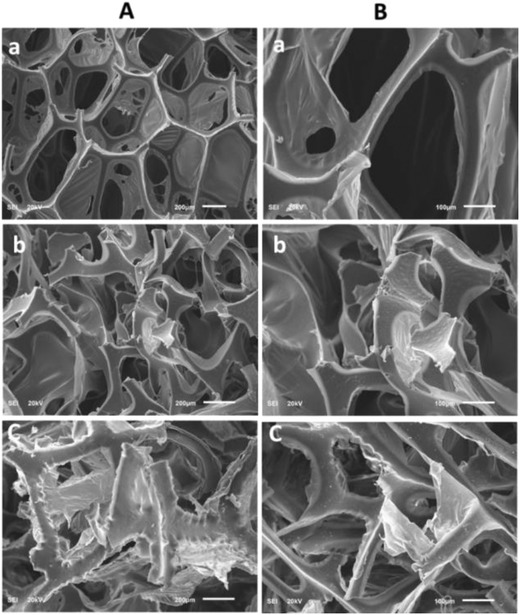
SEM images of a) PU, b) PS/PU, c) 3D zz‐PS/GR/PU at two different magnifications A) 200 and B) 100 µm.

The synthesized materials were characterized by transmission electron microscopy (TEM), scanning electron microscope (SEM), and Fourier transfer infrared spectroscopy (FTIR). The synthesized graphene was characterized by the FTIR and the TEM. The FTIR spectra have shown the characteristic absorption band of aromatic carbon–carbon double bond (—C=C—) at 1633 cm^−1^. During the reduction process of GO, some oxygen‐containing functionalities were retained, which generally appeared in the FTIR spectra of the rGO. The absorption bands of oxygen‐containing functionalities such as carboxy —C—O stretching (ν = 1391 cm^−1^), alkoxy —C—O stretching (ν = 1027 cm^−1^), and hydroxyl group (ν = 3438 cm^−1^) absorption bands appeared in the FTIR spectra of graphene. The TEM study revealed either one or only a few layered graphenes. The FTIR and the TEM study have shown that graphene was successfully synthesized. The typical Raman spectrum of the graphene was determined. The D band appeared at 1350 cm^−1^ and G band appeared at 1580 cm^−1^. The D band was appeared due to the defects in the graphene sheets. The G band was appeared due to the in‐plane vibration of the sp^2^ bonded carbon atoms. In the Raman spectra of the graphene, the intensity of the G band was more compared with the D band, which is an indication of the partial restoration of the conjugation network. The *I*_D_/*I*_G_ ratio was found 0.91 which is less than 1. If *I*_D_/*I*_G_ ratio is large it is an indication that the reduction process generated more defects in the obtained graphene^35^ (**Figure** **2**). The SEM images of the pure PU, PS/PU, and the 3D zz‐PS/GR/PU were recorded at different magnifications to observe the morphological changes on the surface of the materials. The growth of the polystyrene on the surface of the pure PU and GR/PU was seen clearly. The wrinkled shape is observed in the 3D zz‐PS/GR/PU, which could improve the surface area and the mechanical strength of the synthesized material (**Figure** **3**).

**Figure 4:**
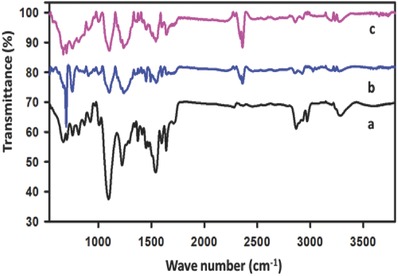
FTIR spectra of a) PU, b) PS/PU, and c) 3D zz‐PS/GR/PU.

The pure polyurethane FTIR spectra (**Figure** **4**a) displayed its characteristics bands.^36^ The vibrational absorption band appeared at 3225–3404 and 1541 cm^−1^ was assigned to —N—H stretching and —N—H deforming, respectively. The —CH_2_ symmetric and asymmetric stretching vibrational bands appeared at 2866 and 2970 cm^−1^, respectively. The absorption band appeared at 1450 cm^−1^ and was assigned to the —CH_2_ bending vibration band. The band appeared at 1714 cm^−1^ in the spectra was assigned to the C=O vibrational band.

In the FTIR spectra of the polyurethane, the asymmetric stretching vibrational band of the NCO at 2270 cm^−1^ was absent. The absence of the absorption band at 2270 cm^−1^ revealed that after polymerization there were no free NCO functionalities.^37^, ^38^ A strong absorption band appeared in the polyurethane spectra at 1097 cm^−1^ was assigned to the stretching of C—O—C.

The introduction of polystyrene and graphene into the polyurethane brought some obvious changes into the FTIR spectra, however, the basic structure of the polyurethane was retained in the composite 3D zz‐PS/GR/PU (Figure 4c). A substantial decrease in the band intensity of the C—O—C was seen after the incorporation of graphene and the styrene polymerization. It was shifted substantially from 1097 (PS/PU) to 1103 cm^−1^ (3D zz‐PS/GR/PU). This interaction of the PU surface with the graphene and the polystyrene might be responsible for the change in the C—O—C band intensity and band position. Apart from this, band shifts in the absorption bands of some functionalities were also observed. For example, a red shift in the —CH_2_ symmetric and asymmetric band of the PS/GR/PU was observed as the bands shifted from 2866 to 2859 and 2970 to 2927 cm^−1^, respectively. A blue shift in the —N‐H deforming band was observed and it was shifted from 1541 to 1542 cm^−1^. The carbonyl band that appeared in the pure polyurethane became considerably unclear in the PS/PU and PS/GR/PU FTIR spectra (Figure 4).

### 2.3 Surface Area and Pore Size Evaluation of 3D zz‐PS/GR/PU

**Figure 5:**
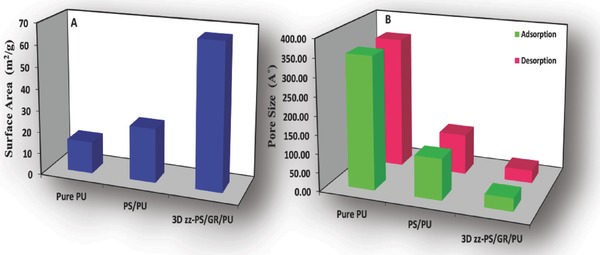
The comparison of the BET A) surface area and B) adsorption and desorption pore size of PU, PS/PU, and 3D zz‐PS/GR/PU.

The utilization of nanomaterials in the synthesis of various composites can bring some extraordinary properties into the material. It is evident that nanomaterials such as graphene have a substantial effect on the surface area of the material.^39^ In the absence of graphene, the pure PU surface area was found to be 15 m^2^ g^−1^. The graphene‐incorporated 3D zz‐PS/GR/PU showed substantial incremental growth in the surface area that increased from 15 to 67 m^2^ g^−1^ (**Figure** **5**A). The graphene and polystyrene also made a great impact on the pore size of the material. The adsorption and desorption pore sizes of the pure PU were found to be 354 and 352 Å, respectively. After the graphene and polystyrene incorporation, the 3D zz‐PS/GR/PU pore size was substantially decreased to 34 and 33 Å (Figure 5B). The incremental growth in the surface area and the decrease in the pore size revealed that there might be new pores and surfaces generated in the 3D zz‐PS/GR/PU, which were responsible for the surface area improvement. Furthermore, the decrease in pore size provided better channels with superhydrophobicity for the efficient separation of oil and water.

### 2.4 Surface Hydrophobicity

**Figure 6:**
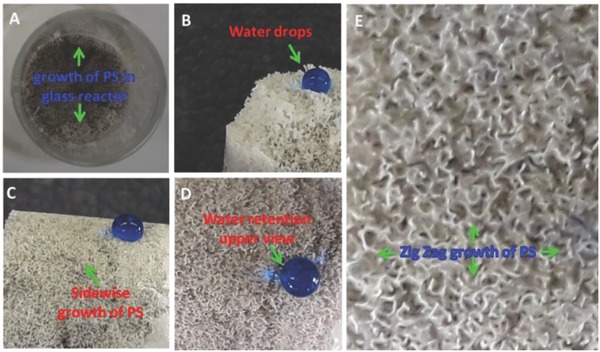
A) Growth of PS into GR/PU in glass reactor, complete retention of water, B) upper side of the zz‐PS/GR/PU, C) sidewise grown of PS, D) upper view of the zz‐PS/GR/PU, and E) magnified image displaying the zigzag formation of PS into GR/PU.

The synthesized 3D zz‐PS/GR/PU hydrophobicity and the oleophilicity were evaluated with the mixture of water and hexane. Both liquids are naturally colorless and methylene blue colored water was used to differentiate between the water and hexane. The growth of the polystyrene is evidently observed from the upper view of the reactor, as seen in **Figure** **6**A. In the start of the reaction process, the styrene liquid was at the bottom of the glass reactor and therefore had no chance to come into direct contact with the GR/PU. The synthesis of the polystyrene on the upper side of the 3D zz‐PS/GR/PU was a clear indication that vapors of the styrene were passing easily through the GR/PU. The passage of vapors all‐around the GR/PU was also evident from the sidewise growth of the polystyrene (Figure 6C). After some detailed and comprehensive investigation of the surface, it was revealed that there was some sort of pattern in the growth of the polystyrene. This pattern of polystyrene was spread all over the 3D composite. These well‐organized patterns appeared in a zigzag arrangement (Figure 6E). This sort of arrangement exposed more surface area and may provide some sort of hollow arrangement that allowed the rapid passage of the nonpolar component. The synthesized composite 3D zz‐PS/GR/PU is superhydrophobic in nature and it can be seen in Figure 6B where the methylene blue colored drop of water was fully retained by the surface. The drop of water became entirely circular on the 3D zz‐PS/GR/PU surface due to the superhydrophobic nature of the surface that did not allow the water to make a significant contact with the surface in order to pass. All sides of the zz‐PS/GR/PU were scanned to observe its behavior toward the water. This study has revealed that all sides of the zz‐PS/GR/PU were superhydrophobic and did not allow the water to spread or pass through it (Figure 6C,D).

**Figure 7:**
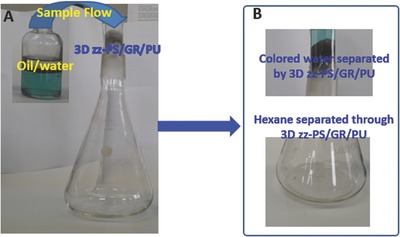
Separation of oil and water. A) Separation setup for oil‐ and water‐containing 3D zz‐PS/GR/PU composite at the neck of the setup, B) The separation of hexane from methylene blue colored water.

**Figure 8:**
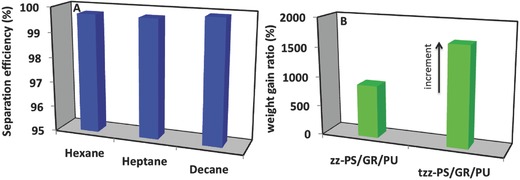
A) Separation efficiency of 3D zz‐PS/GR/PU for nonpolar organic solvents and B) weight gain capacity of the zz‐PS/GR/PU and tzz‐PS/GR/PU.

The established 3D hydrophobic architecture was investigated for its efficiency to separate the oil from water. The separation setup used can be seen in **Figure** **7**A. The water and hexane mixture was added from the upper part of the oil/water separation setup which contained the 3D zz‐PS/GR/PU in its upper part. Amazingly the 3D zz‐PS/GR/PU exhibited both superhydrophobic and superoleophilic behavior. The hexane was allowed to pass quickly simply under the force of gravity without applying any external force, while the methylene blue colored water was not permitted to pass through the 3D zz‐PS/GR/PU. The separation of hexane and water was seen in the separating setup (Figure 7B) where the upper part of the glass reactor contained the methylene blue colored water while the hexane passed through it. The efficiency of the 3D zz‐PS/GR/PU composite for hexane and water separation can be seen in Video S1 in the Supporting Information. In the similar fashion, the hexane and decane were separated from the water. Karl Fischer was helpful in finding the water content in the various separated nonpolar component and gas chromatography with flame ionization detector confirmed the concentration of separated nonpolar organic components. The separation efficiency of the hexane, heptane, and decane was found as 99.5%, 99.7%, and 99.8%, respectively (**Figure** **8**A). The separation efficiency indicated that hydrophobic channels of the 3D zz‐PS/GR/PU are not allowing to pass water because of its high hydrophobicity.

The synthesized 3D zz‐PS/GR/PU composite was not compressible and appeared hard in nature. Due to its noncompressible nature, it exhibited a hard tube‐like capillary behavior which allows for the rapid gravity‐driven passage of hexane. Due to its noncompressible nature, it could not keep a large quantity of the hexane although it still possessed the absorption capability of the nopolar organic solvents. The weight gain ratio of the 3D zz‐PS/GR/PU for hexane was found to be 890%. This is an indication that it could take a good quantity of hexane apart from its compact nature. The absorbed hexane can be released simply by shaking the 3D‐PS/GR/PU due to its weak holding capacity. The synthesized material has long‐term stability and it can be used multiple times without compromising its efficiency for hexane and water separation. It can be shown to be a valuable superhydrophobic material for the bulk separation of oil and water due to the rapid passage of oil through it.

### 2.5 Toluene Treated 3D zz‐PS/GR/PU

**Figure 9:**
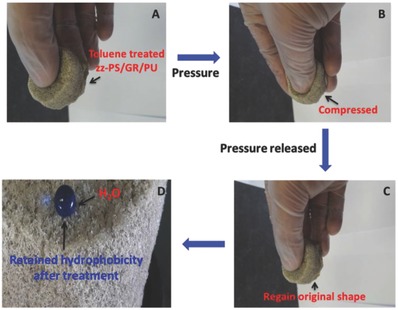
A) Toluene‐treated zz‐PS/GR/PU, B) fully compressed tzz‐PS/GR/PU, C) pressure released on tzz‐PS/GR/PU, and D) superhydrophobicity retained by the tzz‐PS/GR/PU.

As aforementioned, the zz‐PS/GR/PU has a compact and stiff surface that provides tube‐like channels and allows for the rapid passage of the hexane through it. These channels were hydrophobic enough to prevent water from passing. Polystyrene showed some solubility in the toulene.^40^ This interaction of the polystyrene and the toluene was used as a tool to produce the compressible 3D tzz‐PS/GR/PU. The toluene treatment showed some change in the weight of the material. The weight of the 3D tzz‐PS/GR/PU was found to be 8.47% less compared with 3D zz‐PS/GR/PU. This change in weight indicates that some of the polystyrenes dissolved into toluene which broke the continuous structure which imparted the compressible behavior to the material. The compressible nature of the 3D tzz‐PS/GR/PU is shown in **Figure** **9**B and it can be fully pressed between the fingers. After releasing the pressure, it regained its original shape (Figure 9C). This treatment might have some effect on the hydrophobic behavior of the 3D composite. For this reason, the hydrophobic behavior of the 3D tzz‐PS/GR/PU was investigated using the methylene blue colored water. It did not allow the water to pass and the water droplet maintained same circular shape on the 3D tzz‐PS/GR/PU. The polystyrene on the surface somehow became fluffy (Figure 9D). Its capability to absorb hexane was significantly improved and the percent weight gain ratio of the 3D tzz‐PS/GR/PU was improved to 89% compared with 3D zz‐PS/GR/PU (Figure 8B). The absorbed hexane is released from 3D tZZ‐PS/GR/PU by squeezing. The comparison of the synthesized hydrophobic material in term of hexane absorption has shown that their efficiency for hexane absorption is either comparable or superior to the reported hydrophobic materials in **Table** **1**. However, the 3D zz‐PS/GR/PU offers a rapid separation of hexane from water due to its compact superhydrophobic channels which make it superior compared with the other reported materials.

**Table 1:** Comparison of the synthesized hydrophobic materials with other reported hydrophobic material for hexane absorption

Sr#	Adsorbent	BET surface area [m^2^ g^−1^]	Hexane [g g^−1^]	Recycling	Ref.
**1**	Hydrophobic CNF aerogels	18.4	24	Evaporation	41
**2**	Multi‐functional carbon fiber	–	22	Distillation, combustion, squeezing	42
**3**	Polydopamine/chitosan/rGO composite aerogel	51.76	12	Squeezing, heating	43
**4**	Cupric stearate/sponges	–	22.63		44
**5**	Swellable porous PDMS/MWNTs	–	15.05	Squeezing	45
**6**	Graphene aerogels	100–350	25	–	46
**7**	CNT/PDMS‐coated PU sponge		15		47
**8**	MTMS–DMDMS gels	–	6	Squeezing	48
**9**	Al_2_O_3_/PUF foam sponge	–	7	Squeezing	49
**10**	MnO_2_/p(BA‐co‐BMA‐co‐MMA) hybrid resins	74.38	2.07	Drying	50
**11**	**3D zz‐PS/GR/PU**	67	9	Evaporation, shaking,	This work
**12**	**3D tzz‐PS/GR/PU**	–	17	Squeezing, evaporation	This work

## 3 Conclusion

A cost‐effective method is introduced for the fabrication of superhydrophobic 3D zz‐PS/GR/PU material. The 3D superhydrophobic architecture was accomplished by initiating the polymerization of styrene with the help of natural‐sunlight‐containing graphene and polyurethane in a confined glass reactor. The polymerization process produced a well‐decorated zigzag arranged polystyrene pattern on the GR/PU. The incorporation of graphene into PU provided a huge surface area and also mechanical stability to the material. In the graphene‐incorporated PU, the polystyrene patterns and growth were more prominent compared with pure PU. This might be due to the better surface area and the catalytic effect of the graphene. The 3D zz‐PS/GR/PU provided the compact porous superhydrophobic channels for the rapid gravity‐driven separation of oil and water, whereas the 3D tzz‐PS/GR/PU is a compressible material with a high absorbing capability for hexane. The 3D zz‐PS/GR/PU and 3D tzz‐PS/GR/PU showed a hexane absorption capacity of 9 and 17 g g^−1^, respectively. The 3D zz‐PS/GR/PU displayed a high surface area of 67 m^2^ g^−1^ with a small adsorption and desorption pore size of 34 and 33 Å, respectively. The water contact angle displayed by the 3D zz‐PS/GR/PU was approximately 150°. In separated nonpolar components, separation efficiency was found to be >99%. This route of formation can provide a cost‐effective approach to produce a hydrophobic material on a large scale which is generally a challenging task, especially on a laboratory scale. The developed methodology of synthesis is exceedingly robust and reproducible. It may encourage researchers to look for other polymers and nanomaterials for the fabrication of a 3D porous architecture for very demanding applications in the fields of energy, oil/water, supercapacitors, and sensors.

## 4 Experimental Section

*Materials*: Styrene was purchased from the Alfa Aesar. Graphite was acquired from the Fluka. Toluene and hexane were obtained from the Merck and Sigma‐Aldrich, respectively. Ethanol was obtained from the Baker Analyzed Reagent. Commercial polyurethane was purchased from the local market. Distilled water was collected from the laboratory‐based distillation unit.

*Instrumentation*: A MicromeriticsTriStar II Plus instrument was used for the measurement of the surface area and the pore size of the materials. A Goniometer was used for the measurement of the contact angle. SEM images were recorded using JSM‐6610LV scanning electron microscope from JEOL. The FTIR of the materials were collected using a Thermo Scientific Nicolet iS10 instrument. The material drying was done using a Blue M oven. The stirring during fabrication was done with the help of a Thermo‐Scientific magnetic stirrer.

*Synthesis of Graphene*: GO was prepared using a modified Hummers method.^51^ Briefly, the procedure for the synthesis of GO is as follows. In a 500 mL volumetric flask, graphite powder (5 g) was added to 100 mL of concentrated sulfuric acid (H_2_SO_4_) and 100 g of sodium nitrate (NaNO_3_). The resulting solution was stirred for 30 min at 5 °C in an ice‐bath. After that, potassium permanganate (KMnO_4_,) powder (15 g) was added slowly to the flask, and the mixture was heated to 35–40 °C and stirred for another 30 min. Distilled water (200 mL) was then added to the above mixture over a period of 25 min. Finally, 30% H_2_O_2_ was added to the mixture until the formation of a yellowish solid product. The mixture was then kept under stirring. The powder was separated in a centrifuge and washed several times by HCl solution and then by water. The obtained GO was then reduced by sodium. After that, GO was reduced by using ascorbic acid. Around 20 g of ascorbic acid was introduced to the dispersed GO under stirring at 70 °C for 4 h. The obtained material was allowed to cool and then separated in a centrifuge. The obtained reduced graphene was characterized.

*Fabrication of 3D zz‐PS/GR/PU*: A fine dispersion of 0.5 mg mL^−1^ graphene was prepared by sonicating the graphene in ethanol for 1 h. After sonication, a piece of PU was dipped into the ethanol dispersed graphene. After that, it was removed from the ethanol dispersed graphene. It was dried and cured for 12 h in an oven at 60 °C. The obtained graphene‐coated polyurethane was described as GR/PU. After the curing process, the GR/PU was transferred into a glass reactor. The glass reactor contained 3 mL styrene monomer. In the glass reactor, the GR/PU was suspended almost in the middle to prevent it from touching the styrene liquid. Similarly, the PU without graphene coating was also prepared in another glass reactor for performance comparison. The glass reactors contained pure PU and the GR/PU was exposed to the natural sunlight until the styrene liquid disappeared. The zigzag growth of the polystyrene on the GR/PU surface could be noted. After the polystyrene growth, the black color of the GR/PU was changed to blackish white. The achieved architecture of the 3D zigzag polystyrene/graphene‐incorporated polyurethane was described as 3D zz‐PS/GR/PU. The 3D tzz‐PS/GR/PU was obtained by instantly dipping and then removing it from the pure toluene. After this toluene treatment, it was dried at room temperature.

*Experiment for Hexane/Water Separation*: The experimental setup was designed for the hexane and water separation. The glass reactor in which the 3D zz‐PS/GR/PU was synthesized was made open from both ends. It was directly fixed on the opening of the suitable funnel to establish an experimental setup. The hexane and water mixture was prepared by mixing 100 mL hexane and 300 mL water in a reagent bottle. The mixture was introduced through the separation setup. For the absorption experiment, the synthesized material was dipped into hexane and taken out for weight measurement.

## Conflict of Interest

The authors declare no conflict of interest.

## Supporting information

Supplementary
